# Comparative Serum Proteomic Analysis of Different Habitual Coffee Consumption Among Healthy and Obese with and Without Hypertension Groups

**DOI:** 10.3390/cimb48060556

**Published:** 2026-05-25

**Authors:** Jintana Sirivarasai, Sorsia Muttrarak, Prapimporn Chattranukulchai Shantavasinkul, Sittiruk Roytrakul, Waraporn Malilas, Pachara Panpunuan, Piyamitr Sritara

**Affiliations:** 1 Nutrition Unit, Faculty of Medicine Ramathibodi Hospital, Mahidol University, Bangkok 10400, Thailand; 2 Master of Science Program in Nutrition, Faculty of Medicine Ramathibodi Hospital and Institute of Nutrition, Mahidol University, Bangkok 10400, Thailand; sorsia.mu@bgh.co.th; 3 Division of Nutrition and Biochemical Medicine, Department of Medicine, Faculty of Medicine Ramathibodi Hospital, Mahidol University, Bangkok 10400, Thailand; sprapimporn@gmail.com; 4 National Center for Genetic Engineering and Biotechnology (BIOTEC), National Science and Technology Development Agency, Pathum Thani 12120, Thailand; sittiruk@biotec.or.th; 5 Institute of Nutrition, Mahidol University, Nakhon Pathom 73170, Thailand; wamalilas@gmail.com; 6 Division of Cardiology, Department of Medicine, Faculty of Medicine, Ramathibodi Hospital, Mahidol University, Bangkok 10400, Thailand; pachpan@hotmail.com (P.P.); piyamitr.sri@mahidol.ac.th (P.S.)

**Keywords:** coffee intake, proteomics, metabolic status, obesity and hypertension, protein–protein interaction

## Abstract

Coffee consumption has been associated with metabolic and cardiovascular health, but the molecular mechanisms underlying these associations remain unclear. This study investigated the association between coffee intake and circulating proteomic profiles across metabolic conditions using a pooled-serum, exploratory design. Participants were classified into four groups: normal weight (NW), normal weight with coffee intake (NWC), obese with hypertension (OBHT), and obese with hypertension with coffee intake (OBHTC). Differentially expressed proteins (DEPs) were identified using volcano plot criteria (|log_2_FC| ≥ 1, FDR < 0.05), followed by Reactome pathway enrichment, Gene Ontology (GO) molecular function, and Enrichr-derived protein–protein interaction (PPI) analyses. Results: In NW vs. NWC, coffee intake was associated with proteins enriched in receptor-mediated signaling and phosphoinositide pathways. In OBHT vs. OBHTC, DEPs were linked to mitochondrial respiration and oxidoreductase activity. The NW vs. OBHT comparison showed downregulation of metabolic and signaling proteins with enrichment of mitochondrial and stress-response functions. In NWC vs. OBHTC, proteins related to cytokine signaling and vascular function were reduced, while redox-associated regulators were increased. PPI networks highlighted interconnected hubs integrating signaling, metabolism, and immune responses. Conclusion: These findings suggest context-dependent proteomic patterns associated with coffee intake. Given the pooled design and small sample size, results are hypothesis-generating and require validation.

## 1. Introduction

Coffee is one of the most widely consumed beverages in the world, with per capita consumption increasing by 37% over the last two decades (as of 2022). The most significant rises occurred in the Middle East and North Africa (84.2%) and upper middle-income countries (86.1%).with billions of cups drunk daily by people across various populations [1]. Caffeine consumption patterns vary significantly by region and demographic factors, such as age and beverage preference. For example, adolescents in the USA tend to prefer coffee, while tea is more commonly consumed in Asia [2]. Moreover, coffee has attracted significant scientific attention due to its complex chemical composition and potential health implications. Coffee is composed of over 1000 bioactive compounds, including caffeine (methylxanthine), chlorogenic acids (CGA), diterpenes (cafestol and kahweol), trigonelline, and various polyphenolic compounds [3]. Among these, caffeine and CGA are especially important due to their biological activities. CGA has been shown to have antioxidant, anti-inflammatory, neuroprotective, hypolipidemic, and hypoglycemic effects [3].

A non-linear relationship between consumption and health outcomes, with the largest risk reductions occurring at three to four cups per day, has been reported [4]. Relative risks include: all-cause mortality at 0.83, cardiovascular mortality at 0.81, and cardiovascular disease at 0.85. Higher consumption is also associated with a 18% lower cancer risk (relative risk of 0.82) [4]. In contrast, a recent meta-analysis of 15 studies with nearly 298,000 participants found no significant association between high coffee consumption and metabolic syndrome risk (odds ratio [OR]: 0.88; 95% confidence interval [CI]: 0.70–1.10) [5]. Another systematic review and meta-analysis found no significant association between coffee intake and the odds of general obesity (OR: 1.11; 95% CI: 0.92–1.33) or abdominal obesity (OR: 1.03; 95% CI: 0.92–1.15) in adults overall [6]. The association between coffee intake and hypertension (HTN) risk is controversial. Higher coffee consumption is associated with a reduced risk of hypertension in adults. Specifically, cohort studies indicate a 7% reduction, while cross-sectional studies show a greater reduction (21%) in hypertension risk, though results vary by study characteristics [7]. In contrast, a recent meta-analysis (13 longitudinal cohort studies involving a total of 64,650 incident cases of hypertension) showed that coffee intake is not associated with the risk of hypertension [8].

Despite growing interest in the health effects of coffee consumption, there remains a significant gap in understanding its molecular impact across diverse metabolic health statuses, particularly in individuals with coexisting obesity and hypertension, a population at high risk for cardiovascular disease. Previous studies examined plasma proteins using three multiplex proximity extension assays in habitual coffee consumers, showing that two neurology-related proteins, carboxypeptidase M and neutral ceramidase, were significantly increased after coffee intake [9], and an inverse association between coffee intake and plasma leptin and chitinase-3-like protein 1 [10]. While prior studies have examined coffee’s effects on general health markers or single disease conditions, none have comprehensively explored how habitual coffee intake modulates the serum proteome in this specific high-risk group. Moreover, integrating clinical nutrition assessment with a high-throughput omics approach, such as quantitative proteomics, remains underutilized in this context. This study is the first to provide proteomic-level evidence of coffee’s differential biological impact in healthy individuals compared with those with obesity alone or obesity combined with hypertension, thereby addressing a critical gap in both personalized nutrition research and the development of molecular risk-profiling tools for complex metabolic disorders. Therefore, the present study aimed to investigate the associations between habitual coffee intake and circulating serum proteomic profiles among individuals with different metabolic conditions, including normal weight, obesity, and obesity with hypertension, using a quantitative proteomic approach. In addition, the study aimed to identify differential protein expression patterns and biological pathways potentially related to coffee consumption across these metabolic phenotypes.

## 2. Materials and Methods

### 2.1. Participants and Data Collection

This cross-sectional study involved male participants aged between 40 and 60 years (*n* = 101) recruited from the Electricity Generating Authority of Thailand (EGAT) in 2018 as part of the EGAT cohort study. The participants actively engaged in a health survey aimed at identifying risk factors for noncommunicable diseases and underwent comprehensive clinical assessments for laboratory investigations. The methodologies and protocols for the EGAT cohort study and a five-year resurvey are detailed in previous publications [11]. During the visit, demographic information (including age, sex, educational level, smoking, and alcohol consumption), health conditions (specifically, chronic diseases), and prescribed medications were systematically collected. According to the World Health Organization, the Asia-Pacific region defines a body mass index (BMI) of ≥23 kg/m^2^ as overweight and ≥25 kg/m^2^ as obese [12]. In this study, hypertension was defined as a systolic blood pressure (SBP) of 140 mmHg or higher and/or a diastolic blood pressure (DBP) of 90 mmHg or higher, according to the International Society of Hypertension Global Hypertension Practice Guidelines (2020) [13]. For participants with hypertension, the individuals had to be taking a stable dose of antihypertensive agents for a minimum of 6 months.

The participants were stratified into four groups based on metabolic health and habitual coffee consumption: Normal Weight (NW, *n* = 38), Normal Weight with Coffee (NWC, *n* = 24), Obesity with Hypertension (OBHT, *n* = 15), and Obesity with Hypertension and Coffee (OBHTC, *n* = 24). To ensure high internal validity and minimize biological variability, participants were rigorously matched within their respective metabolic categories for age, BMI, and waist circumference (WC). Habitual coffee intake was defined as the consumption of one standard cup (150–200 mL) per day for a minimum of 6 months, with the type of coffee (black coffee versus coffee with sugar and/or non-dairy creamer) further characterized to ensure a homogeneous distribution of exposure between the NWC and OBHTC groups. To mitigate potential confounding from alternative sources of caffeine and polyphenols, we implemented an exclusion criterion for tea consumption. Participants were categorized as non-tea drinkers if their intake was less than once per week.

The exclusion criteria used in this study included the following: intensive diet changes within the last 3 months or a significant weight loss or gain (5%); excessive consumption of energy drinks, high doses of caffeine supplements, or very high tea intake; current heavy smoking (e.g., >10 cigarettes/day) or substance abuse; severe/acute illness, history of acute inflammatory conditions (e.g., active infection, autoimmune), cancer (within 5 years), or severe renal or hepatic disease; chronic or high doses of anti-inflammatory drugs, regular use of oral glucocorticoids (steroids), or high doses of NSAIDs; high doses of supplements (e.g., vitamins/minerals, fish oil/omega-3 supplements, or herbal extracts) or regular use of protein/amino acid supplements.

The research was conducted with the approval of the Committee on Human Rights Related to Research Involving Human Subjects at the Faculty of Medicine, Ramathibodi Hospital, Mahidol University (protocol number: COA. MURA2020/831).

### 2.2. Anthropometric and Clinical Assessment

The BMI was calculated by dividing an individual’s weight in kilograms by the square of their height in meters, providing a measure of body fat relative to height. Waist circumference (WC) was measured while the participant stood upright, taking care to find the midpoint between the lowest ribs and the top edge of the iliac crest. To assess cardiovascular health, systolic blood pressure (SBP) and diastolic blood pressure (DBP) were recorded as the average of two readings. These assessments were conducted using an automatic blood pressure monitor placed on the participant’s left arm, with the participant seated comfortably and having rested for at least 5 minutes to obtain accurate results.

### 2.3. Biochemical Analysis

Venous blood samples were collected systematically in the morning after participants had undergone an overnight fast lasting 12 h. This fasting period was essential to ensure accurate measurements of metabolic parameters. After blood collection, the serum samples were promptly separated and stored at −80 °C to preserve their integrity for future analysis. The measured biochemical parameters included triglycerides (TG), total cholesterol (TC), low-density lipoprotein cholesterol (LDL-C), high-density lipoprotein cholesterol (HDL-C), fasting blood glucose (FBG), hemoglobin A1c (HbA1c), uric acid (UA), blood urea nitrogen (BUN), and creatinine (Cr). These tests were conducted using automated methods on a Cobas-Mira analyzer (Roche, Milan, Italy).

### 2.4. Serum Proteomic Analysis

The proteomic analysis was performed using liquid chromatography/tandem mass spectrometry (LC-MS/MS), as described previously [14]. For preparation and quantification of serum proteins, a pooled sample was prepared for each group, consisting of 20 µg of total serum protein derived from the four groups. The Lowry method was used to accurately determine the total protein concentration in each serum sample prior to pooling [14]. Following this first step, the samples underwent in-gel digestion, which involved embedding the pooled protein samples within a gel matrix. The specific enzyme used for this enzymatic digestion was trypsin, which cleaves peptide chains primarily at the carboxyl side of lysine or arginine residues. The digestion parameters were set to allow up to two missed cleavages to ensure an optimal peptide length distribution for subsequent mass spectrometry analysis. This process converts complex, intact proteins into a mixture of peptides, which are the necessary input for LC-MS/MS acquisition and identification. The resulting peptide mixtures were analyzed following the protocol established in a previous study [15]. The peptides were first separated by LC based on their physicochemical properties. The eluted peptides were then introduced into the mass spectrometer for ionization. In the tandem MS (MS/MS) stage, precursor ions were selected and fragmented, and the resulting fragment ions were mass-analyzed to produce the spectra used for identification.

For protein identification, MaxQuant (v1.6.1.12), integrated with the Andromeda search engine, was used to compare acquired MS/MS spectra against known protein sequences in the UniProt *Homo sapiens* database. The raw MS/MS data were processed in MaxQuant using stringent criteria. The search utilized a mass tolerance of 20 ppm for the main search. Specific modifications were considered, including carbamidomethylation of cysteines as a fixed modification and oxidation of methionine and acetylation of the protein N-terminus as variable modifications. To maintain high confidence in the results, only peptides with a minimum length of seven amino acids and containing at least one unique peptide were accepted. A protein was considered definitively identified and used for subsequent analysis only if it was supported by a minimum of two peptides, with at least one of those being unique to that specific protein.

#### Bioinformatic Analysis of Differential Proteins

Differential protein analysis and downstream functional interpretation were performed using MetaboAnalyst (version 5.0) (https://www.metaboanalyst.ca, accessed on 16 December 2025.) and Enrichr [16,17,18], following established workflows for omics data.

1. Identification of Differentially Expressed Proteins (DEPs). Processed protein abundance data were imported into MetaboAnalyst. Data were log_2_-transformed and normalized (e.g., median or autoscaling, as appropriate) to reduce technical variation and approximate normal distribution. For each pairwise group comparison (NW vs. NWC, OBHT vs. OBHTC, NW vs. OBHT, and NWC vs. OBHTC), differential analysis was conducted using statistical testing implemented in MetaboAnalyst (e.g., Student’s *t*-test). Resulting *p*-values were adjusted for multiple testing using the false discovery rate (FDR, Benjamini–Hochberg method). Proteins were considered significantly differentially expressed based on thresholds of |log_2_ fold change (FC)| ≥ 1.5 and FDR < 0.05. Results were visualized using volcano plots that display both statistical significance and the magnitude of change.

2. Functional Enrichment Analysis. Significant DEPs were exported as gene symbols and uploaded to Enrichr (https://maayanlab.cloud/Enrichr/ (accessed on 16 December 2025)) for functional enrichment analysis. Enrichment was performed using the following:

- Reactome pathway database for biological pathway annotation
- Gene Ontology (GO) Molecular Function for functional characterization

Enrichr calculates enrichment using a combined score integrating Fisher’s exact test *p*-value and a z-score. Terms were ranked based on combined score and statistical significance, and the top enriched terms were selected for interpretation and visualization.

3. Protein–Protein Interaction (PPI) Network Analysis. To explore the interaction context, DEPs were further analyzed using the Enrichr PPI hub protein module, which integrates curated interaction data from multiple databases (e.g., STRING and other repositories). This approach identifies hub proteins with high connectivity and expands the input list by incorporating known or predicted interacting partners, generating a reference interaction network. It is important to note that proteins appearing in the PPI network are not limited to experimentally detected proteins in this study; they also represent database-derived associations.

4. Visualization and Interpretation. Enrichment results were visualized as bar plots (Reactome and GO terms) and clustergrams, illustrating protein–term associations based on gene overlap. The PPI network was used to identify key nodes and interaction patterns, while enrichment analyses provided insight into functional categories and biological processes. All interpretations were conducted with consideration that these analyses are annotation- and database-dependent, thereby reflecting predicted functional relationships rather than direct experimental validation.

### 2.5. Statistical Analysis

Data were analyzed using IBM SPSS version 25 (IBM Corp., Armonk, NY, USA). Normality of continuous variables was assessed using the Shapiro–Wilk test. Continuous variables with non-normal distributions were reported as median and interquartile range (IQR), whereas categorical variables were presented as frequencies and percentages. Differences among the three groups were analyzed using the Kruskal–Wallis test. When a significant difference was detected, post hoc pairwise comparisons were performed using Dunn’s test with Bonferroni correction to adjust for multiple comparisons. Categorical variables, including smoking status, alcohol consumption, medication use, and the intensity of coffee intake, were compared across the four study groups using the Chi-square test or Fisher’s exact test for variables with small expected cell frequencies (*n* < 5). A two-tailed *p*-value < 0.05 was considered statistically significant.

## 3. Results

Table 1 presents the clinical and biochemical characteristics of the four study groups. The median age (57–58 years) and the proportions of current smokers and alcohol consumers were similar across all groups (*p* > 0.05), confirming a well-matched population. The obese/hypertensive groups (OBHT and OBHTC) had significantly higher BMI, waist circumference, blood pressure, HbA1C, and lipid profile compared to the normal-weight groups (NW and NWC) (*p* < 0.001). Inter-group analysis found no significant differences in clinical parameters between coffee drinkers and non-drinkers within the same metabolic category (NMC vs. NW and OBHTC vs. OBHT), indicating that coffee consumption did not affect clinical phenotype or lipid profiles. Regarding medication, 58.26% of the OBHT and 49.32% of the OBHTC groups were on antihypertensive treatment, with similar medication use across the two obese/hypertensive subgroups. This similarity suggests that any differences in the serum proteome are likely due to coffee consumption rather than variations in baseline health.

Coffee consumption patterns varied across the four groups studied. By definition, all participants in the non-coffee groups (NW and OBHT) reported no coffee intake, accounting for 100%. In contrast, among the coffee-consuming groups, the majority indicated moderate consumption of one to two cups per day: 20.8% in the NWC group and 16.7% in the OBHTC group. Higher intake, defined as more than two cups per day, was observed in 79.2% of the NWC participants and 83.3% of the OBHTC participants. Regarding the type of coffee consumed, most participants in both coffee-drinking groups reported drinking black coffee: 70.8% in NWC and 75.0% in OBHTC. A smaller proportion consumed coffee with sugar or cream, which accounted for 29.2% and 25.0%, respectively. Additionally, a significant percentage of participants across all groups reported no tea consumption. The percentages were as follows: 92.1% for NW, 91.7% for NWC, 93.3% for OBHT, and 95.8% for OBHTC.

**Figure 1:**
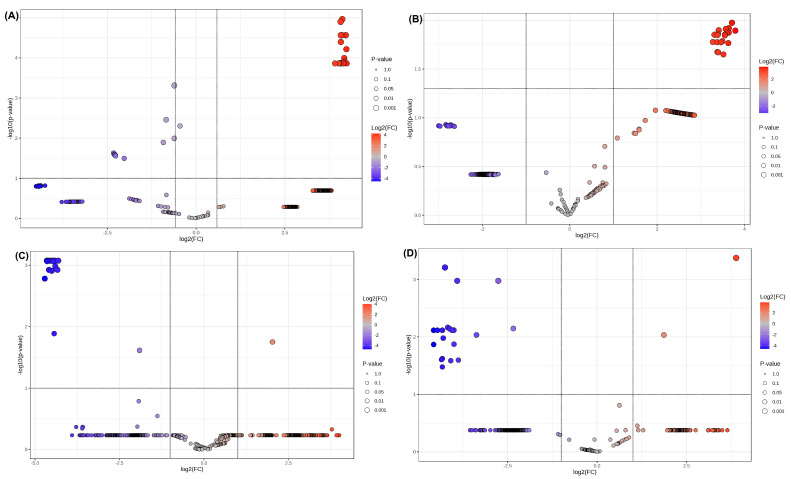
Volcano plots show significant up- and downregulated proteins across four groups: (**A**) NW/NWC, (**B**) OBHT/OBHTC, (**C**) NM/OBHT, and (**D**) NWC/OBHTC. NW, Normal weight group; NWC, Normal weight with coffee; OBHT, Obesity with hypertension; OBHTC, Obesity with hypertension and coffee.

A volcano plot analysis was performed to identify differentially expressed proteins across four pairwise comparisons, using thresholds of log_2_FC ≥ 1.5 and a false discovery rate (FDR) of <0.05 (Figure 1A–D). In the comparison between the NW and NWC groups (Figure 1A), 31 proteins showed significant alterations: 20 were upregulated, and 11 were downregulated in the coffee intake group. This indicates that coffee consumption influences the serum proteomic profile in individuals with normal weight. In the comparison of obese individuals with hypertension (OBHT) versus those with coffee intake (OBHTC) (Figure 1B), 20 proteins were significantly upregulated, whereas none met the criteria for downregulation. This pattern suggests that coffee intake has a predominantly stimulatory effect on protein expression in obese individuals with hypertension. In our analysis comparing the normal-weight (NW) and obese hypertensive (OBHT) groups (Figure 1C), we identified 21 proteins that were differentially expressed, with 1 protein upregulated and 20 proteins downregulated in the OBHT group compared to the NW group. This significant imbalance underscores a potential suppression of protein expression associated with the obese hypertensive condition, indicating an area for further exploration. Similarly, when examining the normal-weight with coffee (NWC) and obese hypertensive with coffee intake (OBHTC) groups (Figure 1D), we identified 24 proteins with significant alterations, including 2 upregulated and 22 downregulated in the OBHTC group relative to the NWC group.

To define the biological significance of the volcano plot findings, differentially expressed proteins were systematically characterized, revealing distinct pathway-level alterations across study groups (Table 2). In the comparison between NW and NWC groups, the upregulated proteins were primarily linked to immune signaling and receptor-mediated pathways, including Proto-oncogene c-Rel (REL), Tumor necrosis factor receptor superfamily member 6 (FAS), Interleukin-1 receptor accessory protein (IL1RAP), and Interleukin-17 receptor E-like protein (IL17REL), indicating an activation of inflammatory and cytokine-related signaling. The concurrent upregulation of Hepatocyte growth factor receptor (MET) and 1-phosphatidylinositol 4,5-bisphosphate phosphodiesterase gamma-1 (PLCG1) further suggests involvement in growth factor signaling and intracellular signal transduction. In contrast, downregulated proteins such as Apolipoprotein A-II (APOA2) and Hemopexin (HPX) indicate changes in lipid transport and antioxidant defense systems. In the comparison between obese hypertensive individuals and obese hypertensive individuals who consume coffee, all 20 significant proteins were upregulated. These proteins were grouped into pathways related to mitochondrial function and oxidative stress response, including NAD(P)H dehydrogenase [quinone] 1 (NQO1), NADH dehydrogenase [ubiquinone] 1 beta subcomplex subunit 10 (NDUFB10), NADH dehydrogenase [ubiquinone] 1 beta subcomplex subunit 11 (NDUFB11), and Cytochrome c1, heme protein, mitochondrial (CYC1), along with regulators of innate immunity and inflammation such as Toll-like receptor 1 (TLR1). Additionally, proteins involved in DNA repair and cellular stress response (Fanconi-associated nuclease 1, FAN1; Bifunctional polynucleotide phosphatase/kinase, PNKP; Splicing regulator SDE2, SDE2) were elevated.

In contrast, the NW vs. OBHT comparison showed a profound shift, with 20 proteins downregulated and only 1 upregulated in the OBHT group. The downregulated proteins were strongly enriched in inflammatory signaling (Interleukin-6 receptor subunit alpha, IL6R; Prostaglandin G/H synthase 2, PTGS2), iron metabolism (Transferrin receptor protein 1, TFRC), lipid metabolism (Mesenteric estrogen-dependent adipogenesis protein, MEDAG), and extracellular matrix remodeling (A disintegrin and metalloproteinase with thrombospondin motifs 7, ADAMTS7), indicating widespread suppression of key physiological pathways. Similarly, in the NWC vs. OBHTC comparison, 24 proteins were significantly altered (2 upregulated, 22 downregulated), with dominant suppression of proteins involved in vascular inflammation and endothelial function (Vascular cell adhesion protein 1, VCAM1; Nitric oxide synthase inducible, NOS2), lipid metabolism and adipokine signaling (Leptin receptor, LEPR; Perilipin-2, PLIN2; Angiopoietin-related protein 3, ANGPTL3), and renin–angiotensin system regulation (Angiotensin-converting enzyme, ACE; Type-1 angiotensin II receptor-associated protein, AGTRAP). Downregulation of oxidative stress-related proteins (NADPH oxidase 4, NOX4) and apoptotic/inflammatory mediators (Caspase-1, CASP1; FAS; Tumor necrosis factor receptor superfamily member 1A; TNFRSF1A) further supports a substantial alteration in cardiometabolic and inflammatory pathways, even in the presence of coffee intake.

**Figure 2:**
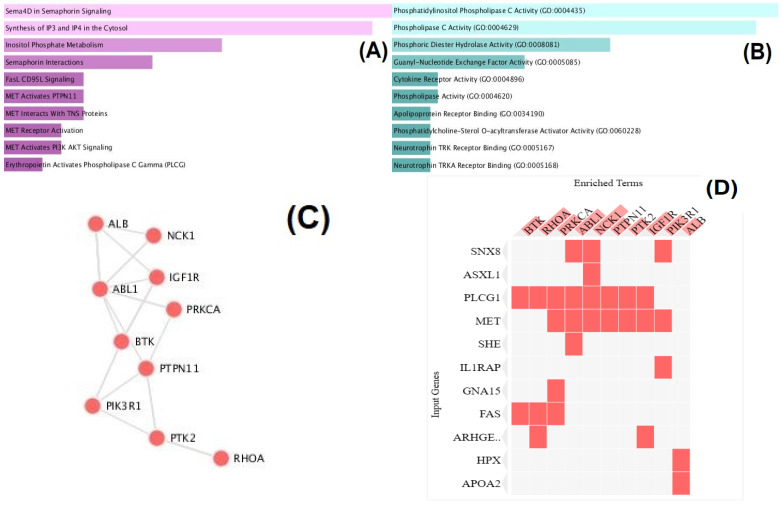
Functional enrichment and protein–protein interaction network of differentially expressed proteins between the Normal weight (NW) and Normal weight with coffee (NWC) groups. (**A**) Reactome pathway enrichment analysis. (**B**) Molecular function enrichment. (**C**) Protein–protein interaction (PPI) network with hub proteins identified by Enrichr. (**D**) Clustergram of enriched proteins. ARHGEF12, Rho guanine nucleotide exchange factor 12.

**Figure 3:**
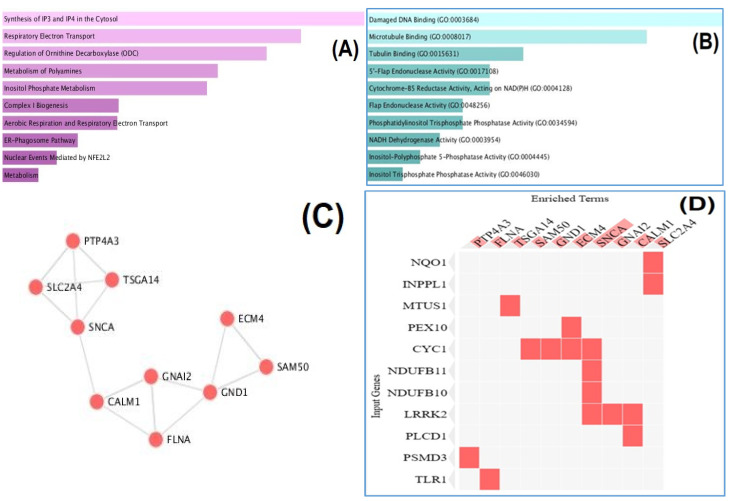
Functional enrichment and protein–protein interaction network of differentially expressed proteins between the OBHT (Obesity with hypertension) and OBHTC (Obesity with hypertension and coffee) groups. (**A**) Reactome pathway enrichment analysis. (**B**) Molecular function enrichment. (**C**) Protein–protein interaction (PPI) network with hub proteins identified by Enrichr. (**D**) Clustergram of enriched proteins.

**Figure 4:**
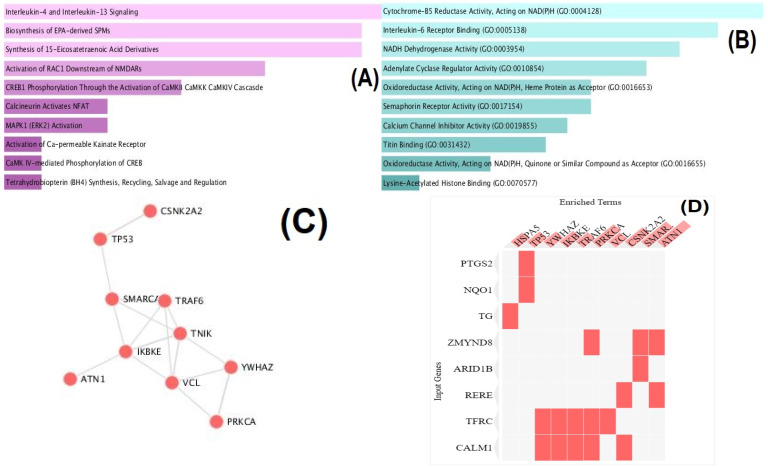
Functional enrichment and protein–protein interaction network of differentially expressed proteins between Normal weight (NW) and Obesity with hypertension (OBHT) groups. (**A**) Reactome pathway enrichment analysis. (**B**) Molecular function enrichment. (**C**) Protein–protein interaction (PPI) network with hub proteins identified by Enrichr. (**D**) Clustergram of enriched proteins.

**Figure 5:**
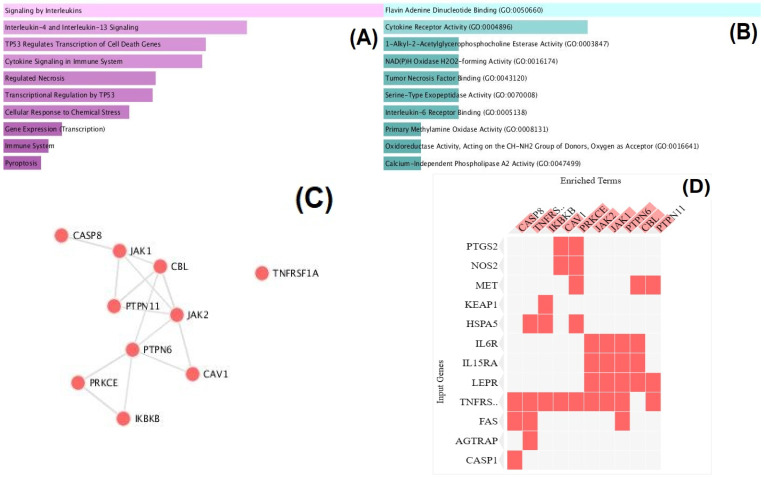
Functional enrichment and protein–protein interaction network of differentially expressed proteins between the Normal weight with coffee (NWC) and Obesity with hypertension and coffee (OBHTC) groups. (**A**) Reactome pathway enrichment analysis. (**B**) Molecular function enrichment. (**C**) Protein–protein interaction (PPI) network with hub proteins identified by Enrichr. (**D**) Clustergram of enriched proteins. TNFRSF1A, Tumor necrosis factor receptor superfamily member 1A.

We analyzed upregulated and downregulated proteins across four comparison groups to identify the top 10 enriched pathways from Reactome and Gene Ontology (GO) Molecular Function, illustrated in Figure 2A,B, Figure 3A,B, Figure 4A,B and Figure 5A,B with detailed statistical analysis in Supplementary Tables S1–S4. Enrichment analysis of differentially expressed proteins (DEPs) between the NW and NWC groups revealed a signaling landscape focused on receptor-mediated signaling and lipid metabolism (Figure 2A,B). Reactome pathway analysis highlighted significant pathways, including Sema4D/Semaphorin signaling and inositol phosphate metabolism, highlighting modulation of intracellular secondary messengers. Additionally, there was notable enrichment of MET receptor signaling and its activation of Protein Tyrosine Phosphatase Non-Receptor Type 11 (PTPN11), phosphatidylinositol 3-kinase (PI3K)/protein kinase B (AKT) (PI3K/AKT), and Phospholipase C-gamma pathways. GO Molecular Function analysis underscored phospholipase C activity and guanyl-nucleotide exchange factor roles. A protein–protein interaction (PPI) network (Figure 2C,D) was generated using Enrichr, expanding the initial protein list to include known and predicted interactions. Key hub proteins identified included Tyrosine-protein kinase ABL1 (ABL1), Focal adhesion kinase 1 (PTK2), and Insulin-like growth factor 1 receptor (IGF1R), with identified proteins like PLCG1 and MET linking to nodes such as Tyrosine-protein kinase BTK (BTK) and Transforming protein RhoA (RHOA). ABL1 showed associations with several proteins, and Phosphoinositide-3-kinase regulatory subunit 1 (PIK3R1) was connected to HPX and APOA2, illustrating interaction patterns within the network and providing an overview of how the proteins relate within known interaction frameworks.

An in-depth analysis of differentially expressed proteins (DEPs) between the OBHT and OBHTC groups was conducted to uncover enriched functional terms and interaction networks. Reactome pathway analysis revealed a significant emphasis on energy metabolism and cellular stress responses, highlighting key pathways such as respiratory electron transport and aerobic respiration (Figure 3A). Additionally, notable enrichment in the synthesis of IP3/IP4 and polyamine metabolism was identified, along with nuclear events mediated by NFE2L2 (Nrf2), suggesting an adaptive response to oxidative stress. GO Molecular Function analysis underscored substantial involvement in genomic integrity, showcasing processes like damaged DNA binding and microtubule binding (Figure 3B). The presence of various inositol phosphatase activities aligns with the Reactome findings, indicating a regulated signaling pathway crucial for metabolic flux, DNA repair, and cytoskeletal organization within the OBHT proteomic landscape.

The PPI network analysis of hub proteins demonstrated a robust interactome among the DEPs (Figure 3C,D). Key nodes such as Calmodulin-1 (CALM1), GND1, and GNAI2 are connected with various partners, forming a dense network that explains the observed proteomic differences. An interaction clustergram mapped DEPs to their identified partners, highlighting 6-phosphogluconate dehydrogenase (GND1) and Glutathione S-transferase omega-like 2 (ECM4) as highly connected nodes. Specific connections were observed between CALM1 and NQO1, as well as Phosphatidylinositol 3,4,5-trisphosphate 5-phosphatase 2 (INPPL1), while Guanine nucleotide-binding protein G(i) subunit alpha-2 (GNAI2) and Alpha-synuclein (SNCA) interacted with 1-phosphatidylinositol 4,5-bisphosphate phosphodiesterase delta-1 (PLCD1) and Leucine-rich repeat serine/threonine-protein kinase 2 (LRRK2), respectively, underscoring the biological significance of the DEPs within a validated network.

The DEPs between the NW and OBHT groups were analyzed using Enrichr to identify enriched functional terms (Figure 4A,B). The Reactome pathway enrichment analysis (Figure 4A) highlighted significant terms, including Interleukin-4 and -13 signaling, eicosapentaenoic acid (EPA)-derived specialized pro-resolving mediators biosynthesis, and Mitogen-Activated Protein Kinase/Extracellular Signal-Regulated Kinase (MAPK1/ERK2) activation. Additional terms such as “Calcineurin activates Nuclear Factor of Activated T cells (NFAT)” and “ Cyclic AMP-Responsive Element-Binding Protein 1 (CREB1) phosphorylation via Calcium/Calmodulin-Dependent Protein Kinase (CaMK) pathways” were also noted. In the molecular function enrichment analysis (Figure 4B), prominent terms included cytochrome-b5 reductase activity, interleukin-6 receptor binding, and NADH dehydrogenase activity. The diverse functional annotations emphasized the varied roles of the identified proteins.

The PPI network analysis revealed a dense cluster of regulatory proteins (Figure 4C), featuring key signaling molecules like Inhibitor of Nuclear Factor Kappa B Kinase Subunit Epsilon (IKBKE), TNF Receptor Associated Factor 6 (TRAF6), and Tumor suppressor p53 (TP53). This indicates that the differentially expressed proteins function through a coordinated signaling module rather than isolated interactions. Figure 4D illustrates associations between DEPs and hub proteins, with major hubs like TP53 and Heat Shock Protein Family A (Hsp70) Member 5 (HSPA5) showing extensive connectivity to proteins such as Transferrin receptor protein 1 (TFRC) and CALM1. Notably, Casein Kinase 2 Alpha 2 (CSNK2A2) and SWI/SNF Related BAF Chromatin Remodeling Complex Subunit ATPase 4 (SMARCA4) were identified as interactors for MYND-type zinc finger-containing chromatin reader (ZMYND8), AT-rich interactive domain-containing protein 1B (ARID1B), and Arginine-glutamic acid dipeptide repeats protein (RERE). This comprehensive analysis lays the groundwork for future investigations into the mechanisms of observed proteomic changes.

The functional enrichment analysis of differentially expressed proteins between the NWC and OBHTC groups revealed significant associations with immune response and inflammation pathways, particularly interleukin-4 and -13 signaling, as well as cytokine signaling (Figure 5A). Additional enrichment was observed in pathways related to cell death and stress responses, including TP53-mediated transcription regulation and regulated necrosis. Molecular function analysis highlighted binding and catalytic activities tied to immune signaling and redox processes, with notable enrichments in flavin adenine dinucleotide binding and cytokine receptor activity (Figure 5B). The PPI analysis identified key hub proteins such as Caspase-8 (CASP8), Janus Kinase 1 (JAK1), Janus Kinase 2 (JAK2), PTPN11, PTPN6, and Inhibitor of Nuclear Factor Kappa-B Kinase Subunit Beta (IKBKB), indicating coordinated regulation in cytokine signaling and apoptotic pathways, while TNFRSF1A appeared more isolated (Figure 5C). The clustering of enriched proteins further emphasized the interconnected relationships among cytokine signaling and immune pathways, as seen in the heatmap pattern, which showed proteins like CASP1 and FAS linked to apoptosis, along with IL6R and TNF receptor-related proteins associated with immune signaling (Figure 5D).

## 4. Discussion

This study reveals a distinct metabolic profile between NW and OBHT individuals, largely independent of coffee consumption. Fasting plasma glucose and HbA1C levels were significantly higher in both OBHT groups compared to NW and NWC, indicating early metabolic dysregulation consistent with compensated insulin resistance, where normal blood sugar levels are maintained despite increased insulin demand [19]. This phase signifies a crucial opportunity to address molecular disturbances that remain undetected by conventional glycemic markers. Lipid profiles further highlight this issue, with OBHT groups showing elevated total cholesterol, LDL, and triglycerides, alongside reduced HDL, creating a pro-atherogenic and lipotoxic environment. This lipid accumulation disrupts insulin signaling through mitochondrial dysfunction and inflammatory responses [20]. The concurrent elevation in triglycerides and blood pressure underscores a cohesive metabolic syndrome phenotype, where cellular stress from lipid overload contributes to insulin signaling impairments before hyperglycemia manifests [21]. The absence of significant biochemical differences between OBHT and OBHTC implies that coffee consumption does not impact routine clinical markers within this cohort. This suggests that dietary factors like coffee can play a valuable role in influencing subclinical processes associated with inflammation, oxidative stress, and metabolism. These processes may not be adequately reflected by general markers, highlighting the need for a broader approach to understanding health [22]. These findings underscore the importance of serum proteomics in elucidating a silent, active state of metabolic dysfunction, highlighting its capacity to identify early alterations that traditional markers may be inadequate to detect. In addition, the proteomic signatures offer valuable insights into the interplay between dietary factors and metabolic stress, facilitating the identification of early biomarkers and potential intervention strategies. Because obesity and hypertension are closely interconnected metabolic conditions, the present study design does not allow complete separation of their individual contributions to the observed proteomic alterations. However, the primary aim of this study was to investigate serum proteomic patterns associated with coffee intake within distinct metabolic phenotypes, particularly in individuals with obesity and hypertension as a combined cardiometabolic condition. The within-group and between-group comparisons were therefore intended to provide comparative insight into how coffee consumption may be associated with proteomic responses under different metabolic contexts rather than to define the isolated molecular effects of obesity or hypertension alone.

In individuals with normal weight, coffee intake was associated with changes in proteins involved in receptor-mediated signaling and phosphoinositide metabolism. Reactome analysis highlighted pathways such as MET receptor activation, FasL/CD95L signaling, and IP3/IP4 synthesis, supported by GO functions like phospholipase C activity and cytokine receptor activity. These findings correlate with the upregulation of proteins such as MET, IL1RAP, PLCG1, and GNA15, reflecting modifications in ligand-receptor interactions and second messenger systems, which are vital for cellular responses to inflammatory and growth signals [23,24]. Additionally, the activation of inositol phosphate metabolism and phospholipase functions suggests involvement in calcium-dependent signaling that regulates vascular tone and immune responses [25]. Semaphorin pathways (e.g., Sema4D signaling) indicate roles in cell communication and immune modulation beyond traditional metabolic pathways [26]. Conversely, downregulated proteins like APOA2, HPX, and ASXL1 relate to lipid transport and oxidative balance, suggesting a shift away from these processes. However, these alterations should be viewed cautiously, as circulating protein levels may reflect complex regulatory dynamics rather than direct suppression.

The PPI network identifies key hub proteins such as PIK3R1, PRKCA, ABL1, and PTPN11, which are involved in PI3K–AKT signaling and adaptor-mediated transduction. Additional hubs, including RHOA, NCK1, and PTK2, link cytoskeletal dynamics to receptor signaling, underscoring the interconnectedness of these pathways [27,28]. The integration of DEPs, enrichment, and PPI analyses indicates that in metabolically normal individuals, coffee intake modulates signaling networks rather than core metabolic pathways, especially those related to phosphoinositide communication and receptor responses. This is consistent with evidence that coffee bioactives can affect cell signaling and inflammatory pathways at a systems level [29].

In individuals with obesity and hypertension, coffee intake (OBHTC) was linked to upregulation of proteins related to mitochondrial metabolism, redox regulation, phosphoinositide signaling, and cellular stress responses. Reactome enrichment indicated enhanced respiratory electron transport, complex I biogenesis, and aerobic respiration, correlating with increased mitochondrial components such as NDUFB10, NDUFB11, and CYC1, as well as NADH dehydrogenase activity in GO terms. Concurrent nuclear events mediated by NFE2L2 and upregulation of NQO1 imply involvement of redox-sensitive antioxidant pathways essential for metabolic adaptation under oxidative stress [30,31]. Enrichment in inositol phosphate metabolism and IP3/IP4 synthesis suggests modulation of second messenger signaling and calcium-dependent pathways, potentially linked to INPPL1 and PLCD1 [25]. Additionally, polyamine metabolism and ornithine decarboxylase regulation point to changes in cellular growth and metabolic regulation associated with mitochondrial function. Upregulated proteins involved in DNA repair (FAN1, PNKP, SDE2) and GO terms like damaged DNA binding indicate genomic maintenance mechanisms, reflecting adaptation to metabolic and oxidative stress [32,33]. The presence of TLR1 and enrichment of the ER-phagosome pathway suggest connections between metabolic processes and inflammation.

The protein–protein interaction (PPI) network identifies key hubs, including SLC2A4 (GLUT4), CALM1, FLNA, and GNAI2, which link glucose metabolism, calcium signaling, and cytoskeletal organization. Additional nodes like SAM50 and SNCA point to mitochondrial integrity and cellular stress responses. These hubs, sourced from curated databases, provide contextual interaction frameworks rather than direct experimental validation. The integration of DEPs, enrichment analyses, and PPI analyses indicates that coffee intake in individuals with obesity and hypertension (OBHT) is associated with the modulation of mitochondrial-redox systems and intracellular signaling pathways, alongside stress-response and immune processes. These findings align with the understanding of cardiometabolic disease, where mitochondrial dysfunction, oxidative stress, and inflammation are interconnected [34,35], though they remain exploratory due to the pooled serum design.

Proteomic comparison of normal-weight (NW) individuals and those with obesity-related hypertension (OBHT) revealed a broad reduction in proteins involved in metabolic, inflammatory, mitochondrial, and signaling regulation, alongside increased ZMYND8, indicating substantial remodeling of the disease proteome [25,30,35,36]. Downregulation of mitochondrial proteins (e.g., NQO1) and enrichment of pathways related to respiratory electron transport and aerobic respiration point to impaired oxidative phosphorylation and altered NADH dehydrogenase activity, consistent with disrupted cellular energy metabolism in obesity and hypertension [30,35,36]. Suppression of serine palmitoyltransferase long chain base subunit 3 (SPTLC3) and Mesenteric Estrogen Dependent Adipogenesis (MEDAG) indicates disturbed lipid biosynthesis and adipocyte function, in line with dysregulated lipid metabolism and altered polyamine/ornithine decarboxylase–related pathways affecting cellular growth and differentiation [32,37]. Decreased IL6R and PTGS2, together with reduced ADAMTS7, CCN2, and PCDH18, suggest attenuated chronic low-grade inflammation and extracellular matrix/cell-adhesion remodeling, potentially reflecting altered receptor availability or compensatory regulation [34,38]. Enrichment of ER–phagosome processes and downregulation of CALM1, along with inositol phosphate and IP3/IP4 pathway changes, indicate impaired calcium-dependent and receptor-coupled signaling relevant to vascular and metabolic control [30,35]. Upregulation of ZMYND8, together with downregulated transcriptional regulators such as ARID1B, and enrichment of damaged DNA-binding and endonuclease activities, support epigenetic remodeling and activation of genomic maintenance pathways under metabolic stress [25,39]. Protein–protein interaction analysis highlights hub proteins CALM1, FLNA, GNAI2, SLC2A4, SNCA, and SAM50, linking cytoskeletal organization, GPCR signaling, glucose transport, and mitochondrial integrity, underscoring that these proteomic alterations are embedded in interconnected networks governing metabolism, signaling, and structural dynamics [35,40,41,42].

Comparison of normal-weight coffee consumers (NWC) and obese hypertensive coffee consumers (OBHTC) revealed a distinct proteomic signature with selective upregulation of metabolic/redox regulators (FABP4, KEAP1) and broad downregulation of proteins involved in immune signaling, vascular function, and cell death. Integrated Reactome, GO, and PPI analyses converged on interconnected modules governing inflammatory signaling, oxidative stress, and programmed cell death, indicating a disease-associated network that persists despite coffee intake [31,34,43]. Reactome enrichment highlighted signaling by interleukins, cytokine signaling in the immune system, and IL-4/IL-13 pathways, consistent with reduced levels of IL6R, IL15RA, TNFRSF1A, and TNFRSF6 and GO terms related to cytokine receptor activity and TNF binding, suggesting altered ligand–receptor immune interactions that may reflect receptor downregulation or compensatory immune modulation rather than simple attenuation of inflammation [34,43]. Enrichment of TP53-regulated transcription, regulated necrosis, and pyroptosis, together with reduced CASP1, CYCS, and COX4I1, indicates remodeling of mitochondrial and non-apoptotic cell death pathways, while decreased NOS2 and NOX4, supported by NAD(P)H oxidase–related GO terms, points to reprogrammed ROS signaling and stress responses [44,45]. Upregulated KEAP1 and FABP4, coupled with downregulated FSP1 and PTGS2 and oxidoreductase-related GO enrichment, underscore redox-sensitive lipid metabolism and ferroptosis-related pathways as central features in OBHTC [31,44,45,46]. Coordinated downregulation of LEPR, ACE, MET, VCAM1, and ANGPTL3 suggests persistent disruption of cardiometabolic and vascular signaling despite coffee consumption, consistent with structural and signaling alterations in obesity-related hypertension [47,48]. PPI network analysis identified hub nodes (JAK1, JAK2, PTPN6, PTPN11, IKBKB, PRKCE, CAV1, CBL, CASP8) anchoring these changes within dense cytokine, NF-κB, membrane, and apoptotic signaling frameworks, emphasizing the systems-level integration of immune, inflammatory, and stress-response dysregulation in OBHTC [49,50,51].

When integrating all four comparisons, this exploratory, pooled-serum proteomic analysis suggests that coffee intake interacts with metabolic status related to circulating protein networks in a context-dependent manner. In normal-weight individuals, coffee consumption was associated with enrichment of receptor-mediated signaling and phosphoinositide pathways (e.g., MET and Fas signaling, IP3/IP4 metabolism), with molecular functions such as phospholipase and cytokine receptor activity and PPI hubs including ABL1, PTPN11, and PRKCA, consistent with prior evidence that coffee bioactives modulate cell signaling and inflammation [52,53]. In contrast, in obese hypertensive participants, coffee intake was linked to proteins involved in mitochondrial respiration and oxidoreductase activity (e.g., NQO1, electron transport chain components) and hubs such as PIK3R1, PTK2, and ABL1, aligning with reports that coffee may engage redox-balancing and metabolic stress pathways [29,54]. Independent of coffee intake, obesity with hypertension was characterized by coordinated downregulation of mitochondrial, metabolic, and signaling proteins, enrichment of oxidative phosphorylation, inositol phosphate metabolism, and DNA damage-related functions, and key hubs such as SLC2A4, CALM1, and FLNA, in agreement with current models of cardiometabolic dysfunction involving impaired glucose transport, altered calcium signaling, and cytoskeletal dysregulation [34,36]. Moreover, comparison of normal-weight and obese hypertensive coffee consumers indicated reduced cytokine receptors and vascular-related proteins (IL6R, TNFRSF1A, ACE, VCAM1), together with enrichment of interleukin signaling, TP53-regulated transcription, regulated cell death, and redox-sensitive regulators (e.g., KEAP1, FABP4) and hubs such as JAK1/2, IKBKB, and PTPN6, suggesting that immune–stress and vascular networks remain perturbed in obesity-related hypertension despite coffee exposure [29,31,34,36,43,44,45,46,47,48,52,53,54].

Furthermore, our findings indicated p a pivotal role of PI3K–AKT pathway in obesity and insulin resistance, as it is essential for the regulation of normal metabolic processes. Dysfunction within this pathway results in insulin resistance, which is a defining characteristic of obesity and type 2 diabetes [55]. This insulin resistance further exacerbates the impairment of the PI3K–AKT pathway, establishing a detrimental cycle that leads to increased metabolic dysregulation [55]. In individuals with concurrent obesity and hypertension, the hyperactivation of the LEPR and MET pathways, driven by increased levels of leptin and growth factors, leads to chronic signaling through the PI3K–AKT pathway [56]. This chronic activation results in systemic insulin resistance. At the same time, the angiotensin II generated by ACE and IL-6 receptor (IL6-R) signaling through the PI3K–AKT pathway contributes to vascular hypertrophy and pro-inflammatory processes, which in turn raise blood pressure and increase vascular stress [57,58]. Additionally, NOX4 produces excessive reactive oxygen species (ROS) in response to angiotensin II and IL-6, further oxidizing the PI3K–AKT pathway and reducing levels of nitric oxide [59]. This interconnected signaling network directly links metabolic fat accumulation to endothelial dysfunction, accelerating both obesity-related health issues and progressive hypertension.

There are certain limitations that must be taken into account when utilizing the outcome. The sample size in each group was relatively small. This study used comprehensive serum proteomic profiling to analyze protein expression patterns linked to coffee intake among normal-weight individuals, obese individuals, and obese individuals with hypertension. We recognize that the limited sample size may reduce statistical power and increase the likelihood that some of the observed differences in proteomics could be due to random variation rather than genuine biological effects. However, several proteins and pathways identified in the study were biologically relevant and aligned with mechanisms related to metabolic dysfunction, inflammation, and cardiovascular regulation. These findings provide valuable preliminary molecular insights that should be further validated in larger, independent cohorts. In addition, the use of pooled serum samples limited the ability to perform individual-level statistical analyses, including evaluating inter-individual consistency, adjusting for potential confounding variables, and correlating protein abundance with clinical parameters such as BMI, blood pressure, glucose, lipid profile, and inflammatory markers. Nevertheless, the pooling strategy was deliberately implemented to capture representative group-level proteomic patterns while minimizing the influence of extreme individual variability that could obscure biologically meaningful signals in comparative analyses. Consequently, the findings should be interpreted primarily at the group-comparison level rather than as individualized molecular associations. Importantly, several identified proteins and pathways were closely related to established mechanisms involved in obesity, hypertension, inflammation, and metabolic regulation, supporting the biological relevance of the observed proteomic alterations. Future studies using individual-level proteomic approaches and larger cohorts are warranted to further validate these findings and enable more comprehensive clinicoproteomic analyses.

In addition, the present study provides an initial characterization of serum proteomic patterns associated with coffee intake across different metabolic conditions rather than to establish definitive molecular effects or causal relationships. Nevertheless, the observed proteomic alterations may offer useful insights for future mechanistic investigations and biomarker-focused studies related to coffee consumption, obesity, and hypertension. The observational nature of the study limits the ability to establish causal relationships between coffee intake and the observed proteomic alterations. We acknowledge that differences between coffee consumers and non-consumers may also be influenced by other dietary habits, lifestyle behaviors, or clinical factors that could not be entirely controlled. Nevertheless, the study provides valuable molecular profiling data across different metabolic phenotypes and may contribute to future controlled intervention and mechanistic studies aimed at clarifying the specific effects of coffee consumption on serum proteomic patterns. Coffee consumption in this study was evaluated using a dietary assessment approach designed to capture regular intake patterns in daily living conditions, with participants classified according to consistent long-term coffee consumption behavior. Additional efforts were made to reduce heterogeneity of exposure by considering major coffee consumption patterns and minimizing potential interference from other caffeine-containing beverages. However, we acknowledge that certain variables, including brewing techniques, variability in caffeine concentration across coffee products, and the interval between coffee intake and blood collection, were not fully standardized. These factors are difficult to control in population-based nutritional studies due to differences in individual consumption habits and commercially available coffee preparations, and they may contribute to variability in circulating proteomic profiles. Future studies with more detailed exposure standardization are warranted. For our study, multiple demographic, clinical, and lifestyle factors may influence circulating serum proteomic profiles. To minimize potential confounding effects, the present study was conducted exclusively in male participants, and the mean age did not significantly differ among the study groups. In addition, coffee consumer and non-consumer subgroups within each metabolic condition were comparable in major metabolic parameters. As expected, differences in BMI, lipid profile, and blood glucose were primarily observed between normal-weight and obese/hypertensive groups, reflecting the underlying metabolic characteristics of the study population. Nevertheless, we recognize that residual confounding from other lifestyle and clinical variables cannot be completely excluded due to the observational design of the study. Future studies involving larger and more diverse populations, comprehensive lifestyle assessments, and controlled intervention designs are warranted to further clarify the independent effects of coffee consumption on serum proteomic profiles. For bioinformatic analyses, Reactome, Gene Ontology (GO), Enrichr, and protein–protein interaction (PPI) network analyses are primarily predictive and intended to support biological interpretation rather than provide direct evidence of pathway activation or inhibition. In the present study, these approaches were applied to identify potential functional relationships and molecular patterns associated with coffee intake across different metabolic phenotypes. Therefore, the findings should be interpreted as hypothesis-supporting rather than definitive mechanistic evidence. Nevertheless, several enriched pathways were biologically plausible and consistent with known mechanisms related to metabolic regulation, inflammation, and cardiovascular function. Future studies should incorporate targeted functional validation and mechanistic experiments to confirm the biological activity of the identified pathways.

## 5. Conclusions

This study demonstrates that coffee intake is associated with distinct circulating proteomic patterns across different metabolic phenotypes, particularly involving receptor signaling pathways in normal-weight individuals and redox–metabolic regulation in obese individuals with hypertension. In contrast, obesity- and hypertension-related alterations in immune and vascular signaling appeared to persist despite coffee consumption, suggesting that underlying cardiometabolic disturbances may substantially influence proteomic responses to dietary exposure. Although these findings provide novel molecular insights into the relationship between coffee intake and metabolic health, further validation in larger individual-level cohorts is required to confirm the observed proteomic alterations and their biological significance.

## Figures and Tables

**Table 1:** Demographic characteristics and biochemical parameters of the study group (*n* = 101).

Characteristic	NM Group	NWC Group	OBHT Group	OBHTC Group
	(*n* = 38)	(*n* = 24)	(*n* = 15)	(*n* = 24)
Age (years)	57.5 (57–59)	58.0 (57–290	58.0 (57–59)	58.0 (57–59)
Current smoker (%) *	7.9	5.9	14	15.1
Current drinker (%) *	51.1	59.6	59.4	54
BMI (kg/m^2^)	21.5 (20.6–22.2)	21.8 (20.8–22.4)	27.3 (25.9–29.1) ^a,b^	27.4 (26.1–30.2) ^a,b^
Waist circumference (cm)	83.2 (79.0–87.5)	84.50 (80.0–86.8)	97.6 (94.0–104.1) ^a,b^	99.2 (95.0–105.5) ^a,b^
Systolic BP (mmHg)	132 (112–149)	131 (122–137)	143 (137–153) ^a,b^	145 (136–151) ^a,b^
Diastolic BP (mmHg)	76 (71–83)	76 (69–80)	88 (81–96) ^a,b^	86 (81–91) ^a,b^
Fasting glucose (mg/dL)	95.0 (85.0–101.2)	92.0 (86.0–96.0)	98.00 (99.00–111.00)	98.0 (90.0–117.0)
HbA1C (%)	5.6 5 (5.40–5.80)	5.5 (5.30–5.80)	6.15 (5.55–6.75) ^a,b^	6.26 (5.81–6.71) ^a,b^
Total cholesterol (mg/dL)	203 (176–222)	203 (176–242)	225 (196–268) ^a,b^	222 (191–250) ^a,b^
LDL-cholesterol (mg/dL)	123.1 (103.2–130.1)	124.3 (89.1–135.2)	139.5 (109.0–158.2) ^a,b^	138.01 (106.2–172.0) ^a,b^
HDL-cholesterol (mg/dL)	57.0 (49.0–66.0)	57 (46.5–67.5)	48.0 (42.0–55.0) ^a,b^	48.9 (42.0–68.2) ^a,b^
Triglyceride (mg/dL)	101.5 (78.0–148.7)	103.0 (73.5–172.5)	149.0 (106.0–195.0) ^a,b^	130.0 (10.70–203.0) ^a,b^
Blood urea nitrogen (mg/dL)	13.3 (11.4–15.1)	12.8 (11.1–13.3)	13.5 (11.5–15.5)	13.4 (11.0–15.0)
Creatinine (mg/dL)	0.9 (0.8–1.1)	0.9 (0.9–1.1)	1.0 (0.9–1.1)	1.0 (0.9–1.1)
Aspartate aminotransferase (U/L)	22 (19–27)	21 (18–24)	23 (19–27)	22 (20–43)
Alanine aminotransferase(U/L)	21 (16–29)	20 (15–23)	25(19–34)	26 (20–37)
Antihypertensive medication (%)	-	-	58.2	49.3
Coffee intake (%) *				
Non-coffee drinker	100	-	100	-
1–2 cups/day	-	20.8	-	16.7
>2 cups/day	-	79.2	-	83.3
Type of coffee consumption (%) *				
-Black coffee	-	70.8	-	75.0
-With sugar/creamer	-	29.2	-	25.0
No-tea drinker (%) *	92.1	91.7	93.3	95.8

Data are expressed as median (interquartile range) or as frequency in percentage. Comparisons between groups were analyzed using the Kruskal–Wallis test. ^a,b^ Significant differences from NW and NWC groups, respectively (Dunn’s post hoc test with Bonferroni correction, *p* < 0.05). * Categorical variables are expressed as percentages (%) and were compared using the Chi-square test or Fisher’s exact test, as appropriate. BMI, body mass index; BP, blood pressure; HbA1C, glycated hemoglobin; HDL-C, high-density lipoprotein cholesterol; NW, Normal weight group; NWC, Normal weight with coffee; OBHT, Obesity with hypertension; OBHTC, Obesity with hypertension and coffee.

**Table 2:** Differential protein abundance across study groups identified via MetaboAnalyst -volcano plot analysis.

	Protein ID	Protein Name	Gene Name	Log 2 FC	FDR
**Group 1: NW/NWC**					
**Upregulated Proteins**					
1	Q04864	Proto-oncogene c-Rel	REL	4.118	0.001
2	P25445	Tumor necrosis factor receptor superfamily member 6	FAS	4.073	0.001
3	P11245	Arylamine N-acetyltransferase 2	NAT2	4.216	0.001
4	Q7Z3B3	KAT8 regulatory NSL complex subunit 1	KANSL1	4.216	0.001
5	Q9Y2B4	TP53-target gene 5 protein	TP53TG5	4.216	0.001
6	Q9Y5X2	Sorting nexin-8	SNX8	4.216	0.001
7	P58743	Prestin	SLC26A5	4.103	0.001
8	P54922	ADP-ribosylhydrolase ARH1	ADPRH	4.077	0.001
9	Q96DB5	Regulator of microtubule dynamics protein 1	RMDN1	4.077	0.001
10	P30679	Guanine nucleotide-binding protein subunit alpha-15	GNA15	4.079	0.001
11	O75354	Ectonucleoside triphosphate diphosphohydrolase 6	ENTPD6	4.174	0.001
12	Q4LE39	AT-rich interactive domain-containing protein 4B	ARID4B	4.174	0.001
13	Q9NPH3	Interleukin-1 receptor accessory protein	IL1RAP	4.174	0.001
14	P08581	Hepatocyte growth factor receptor	MET	4.185	0.001
15	Q6ZVW7	Interleukin-17 receptor E-like protein	IL17REL	4.185	0.001
16	Q9ULI0	ATPase family AAA domain-containing protein 2B	ATAD2B	4.185	0.001
17	Q5VT25	Serine/threonine-protein kinase MRCK alpha	CDC42BPA	4.145	0.001
18	Q9NYL5	24-hydroxycholesterol 7-alpha-hydroxylase	CYP39A1	4.145	0.001
19	P19174	1-phosphatidylinositol 4,5-bisphosphate phosphodiesterase gamma-1	PLCG1	4.059	0.001
20	Q8IWE2	Protein NOXP20	FAM114A1	4.059	0.001
**Downregulated proteins**					
1	Q6ZQQ6	WD repeat-containing protein 87	WDR87	−0.845	0.001
2	A8K010	Putative transcriptional regulator encoded by LINC00473	LINC00473	−0.618	0.026
3	P02652	Apolipoprotein A-II	APOA2	−2.329	0.001
4	Q5VZ18	SH2 domain-containing adapter protein E	SHE	−2.329	0.001
5	Q9NZN5	Rho guanine nucleotide exchange factor 12	ARHGEF12	−2.329	0.001
6	P02790	Hemopexin	HPX	−2.299	0.001
7	Q9P212	1-phosphatidylinositol 4,5-bisphosphate phosphodiesterase epsilon-1	PLCE1	−2.299	0.001
8	P19835	Bile salt-activated lipase	CEL	−2.300	0.001
9	Q8IXJ9	Polycomb group protein ASXL1	ASXL1	−2.300	0.001
10	A6NC57	Ankyrin repeat domain-containing protein 62	ANKRD62	−2.296	0.001
11	Q07075	Glutamyl aminopeptidase	ENPEP	−2.296	0.001
**Group 2: OBHT/OBHTC**					
**Upregulated proteins**					
1	O60683	Peroxisome biogenesis factor 10	PEX10	3.790	0.015
2	Q6IQ49	Splicing regulator SDE2	SDE2	3.770	0.016
3	Q9Y2M0	Fanconi-associated nuclease 1	FAN1	3.750	0.016
4	O15357	Phosphatidylinositol 3,4,5-trisphosphate 5-phosphatase 2	INPPL1	3.713	0.015
5	Q5S007	Leucine-rich repeat serine/threonine-protein kinase 2	LRRK2	3.709	0.015
6	Q9UKA1	F-box/LRR-repeat protein 5 (F-box and leucine-rich repeat protein 5)	FBXL5	3.704	0.015
7	P15559	NAD(P)H dehydrogenase [quinone] 1	NQO1	3.684	0.015
8	Q8NG31	Outer kinetochore KNL1 complex subunit KNL1	KNL1	3.655	0.015
9	A3KMH1	von Willebrand factor A domain-containing protein 8	VWA8	3.659	0.016
10	Q15399	Toll-like receptor 1 (Toll/interleukin-1 receptor-like protein)	TLR1	3.644	0.016
11	Q9NX14	NADH dehydrogenase [ubiquinone] 1 beta subcomplex subunit 11	NDUFB11	3.639	0.016
12	P51178	1-phosphatidylinositol 4,5-bisphosphate phosphodiesterase delta-1	PLCD1	3.629	0.017
13	Q96T60	Bifunctional polynucleotide phosphatase/kinase	PNKP	3.627	0.017
14	O96000	NADH dehydrogenase [ubiquinone] 1 beta subcomplex subunit 10	NDUFB10	3.591	0.016
15	Q6ZMV8	Putative zinc finger protein 730	ZNF730	3.588	0.016
16	O43242	26S proteasome non-ATPase regulatory subunit 3	PSMD3	3.572	0.015
17	Q5T7N2	LINE-1 type transposase domain-containing protein 1	L1TD1	3.565	0.015
18	Q9ULD2	Microtubule-associated tumor suppressor 1	MTUS1	3.600	0.015
19	P08574	Cytochrome c1, heme protein, mitochondrial	CYC1	3.552	0.016
20	Q8IZF5	Adhesion G-protein coupled receptor F3	ADGRF3	3.511	0.016
**Group 3: NW/OBHT**					
**Upregulated proteins**					
1	Q9BXR5	MYND-type zinc finger-containing chromatin reader	ZMYND8	2.209	0.001
**Downregulated proteins**					
1	ARID1B	AT-rich interactive domain-containing protein 1B	ARID1B	−4.650	0.001
2	NQO1	NAD(P)H dehydrogenase [quinone] 1	NQO1	−4.621	0.001
3	SPTLC3	Serine palmitoyltransferase 3	SPTLC3	−4.621	0.001
4	IGKV1–13	Immunoglobulin kappa variable 1–13	IGKV1–13	−4.604	0.001
5	PCDH18	Protocadherin-18	PCDH18	−4.604	0.001
6	TFRC	Transferrin receptor protein 1	TFRC	−4.589	0.001
7	ZNF416	Zinc finger protein 416	ZNF416	−4.589	0.001
8	MEDAG	Mesenteric estrogen-dependent adipogenesis protein	MEDAG	−4.557	0.001
9	PTGS2	Prostaglandin G/H synthase 2	PTGS2	−4.510	0.001
10	ADAMTS7	A disintegrin and metalloproteinase with thrombospondin motifs 7	ADAMTS7	−4.510	0.001
11	TG	Thyroglobulin	TG	−4.460	0.001
12	NOL10	Nucleolar protein 10	NOL10	−4.460	0.001
13	CCN2	CCN family member 2	CCN2	−4.455	0.001
14	RERE	Arginine-glutamic acid dipeptide repeats protein	RERE	−4.455	0.001
15	IL6R	Interleukin-6 receptor subunit alpha	IL6R	−4.428	0.001
16	LINC00574	Putative uncharacterized protein LINC00574	LINC00574	−4.428	0.001
17	CALM1	Calmodulin-1	CALM1	−4.405	0.001
18	PLXNA4	Plexin-A4	PLXNA4	−4.405	0.001
19	GRAMD4	GRAM domain-containing protein 4	GRAMD4	−4.313	0.001
20	DBH	Dopamine beta-hydroxylase	DBH	−4.640	0.001
**Group 4: NWC/OBHTC**					
**Upregulated proteins**					
1	P15090	Fatty acid-binding protein, adipocyte	FABP4	3.884	0.001
2	Q14145	Kelch-like ECH-associated protein 1	KEAP1	1.865	0.009
**Downregulated proteins**					
1	P11021	Endoplasmic reticulum chaperone BiP	HSPA5	−4.565	0.008
2	Q9Y5C1	Angiopoietin-related protein 3	ANGPTL3	−4.565	0.014
3	Q9BRK3	Matrix remodeling-associated protein 8	MXRA8	−4.459	0.008
4	Q99541	Perilipin-2	PLIN2	−4.346	0.025
5	P48357	Leptin receptor	LEPR	−4.322	0.008
6	P08581	Hepatocyte growth factor receptor	MET	−4.322	0.024
7	Q9NPH5	NADPH oxidase 4	NOX4	−4.322	0.033
8	Q13093	Platelet-activating factor acetylhydrolase	PLA2G7	−4.298	0.011
9	Q9HCL0	Protocadherin-18	PCDH18	−4.248	0.001
10	P35228	Nitric oxide synthase, inducible	NOS2	−4.248	0.001
11	Q13261	Interleukin-15 receptor subunit alpha	IL15RA	−4.248	0.001
12	P19320	Vascular cell adhesion protein 1	VCAM1	−4.170	0.007
13	P29466	Caspase-1	CASP1	−4.116	0.007
14	P13073	Cytochrome c oxidase subunit 4 isoform 1, mitochondrial	COX4I1	−4.088	0.026
15	P99999	Cytochrome c	CYCS	−4.050	0.008
16	P08887	Interleukin-6 receptor subunit alpha	IL6R	−4.000	0.008
17	Q9BRQ8	Ferroptosis suppressor protein 1	AIFM2	−3.997	0.013
18	P35354	Prostaglandin G/H synthase 2	PTGS2	−3.907	0.001
19	P19438	Tumor necrosis factor receptor superfamily member 1A	TNFRSF1A	−3.875	0.025
20	P25445	Tumor necrosis factor receptor superfamily member 6	FAS	−3.369	0.009
21	P12821	Angiotensin-converting enzyme	ACE	−2.761	0.001
22	Q6RW13	Type-1 angiotensin II receptor-associated protein	AGTRAP	−2.340	0.007

Abbreviation: NW, Normal weight group; NWC, Normal weight with coffee; OBHT, Obesity with hypertension; OBHTC, Obesity with hypertension and coffee.

## Data Availability

The original contributions presented in this study are included in the article/Supplementary Material. Further inquiries can be directed to the corresponding author.

## Supplementary Materials

The following supporting information can be downloaded at: https://www.mdpi.com/article/10.3390/cimb48060556/s1.
